# What is known about Cycling Without Age: A Scoping Literature Review

**DOI:** 10.1093/geroni/igab046.3204

**Published:** 2021-12-17

**Authors:** Nicole Kaufman, Aleksandra Zecevic, Suna Christensen, Cotnam Victoria

**Affiliations:** 1 University of Western Ontario, Toronto, Ontario, Canada; 2 Western University, London, Ontario, Canada; 3 Independent Researcher, Copenhagen, Hovedstaden, Denmark; 4 Western University, Western University, Ontario, Canada

## Abstract

The Cycling Without Age (CWA) program provides residents of long-term care homes with a bike ride experience, as a volunteer pedals them around the community in a specially designed trishaw. There is limited evidence of the program's effectiveness on older adults, pilots, and communities. The purpose of this literature review is to scope and summarize contemporary CWA discourses to generate future research questions that will provide evidence for future implementation of CWA. Data collection and analysis followed Arksey and O'Malley's 2005 framework. A systematic search was conducted in PubMed, OMNI, and Ebscohost databases. A grey literature search strategy incorporated: grey literature databases, customized Google searches, targeted websites, consultation with expert librarians, and a social media analysis on Twitter, Facebook and LinkedIn. Content analysis was used to identify the key themes. A total of 165 sources (2 peer-reviewed, 103 grey literature, 60 social media) were included in the final analysis. The three main themes were (a) meaning from being on a bike, (2) impacts of CWA, and (3) formation of relationships. Findings suggest that the CWA program brought valuable meaning to the participants' lives, significantly improved their happiness, and was associated with the formation of new and diverse intergenerational relationships. A large amount of anecdotal evidence, social media chatter, and global adoption of CWA indicate its importance and potential to satisfy the need of older adults to engage with society. Future research on the physical and mental health benefits of CWA is required to support further implementation of the program.

